# An Evaluation of the British Columbia Asthma Monitoring System (BCAMS) and PM_2.5_ Exposure Metrics during the 2014 Forest Fire Season

**DOI:** 10.3390/ijerph120606710

**Published:** 2015-06-12

**Authors:** Kathleen E. McLean, Jiayun Yao, Sarah B. Henderson

**Affiliations:** 1 Environmental Health Services, British Columbia Centre for Disease Control, 655 West 12th Avenue, Vancouver, BC V5Z 4R4, Canada; E-Mails: kathleen.mclean@bccdc.ca (K.E.M.); jiayun.yao@bccdc.ca (J.Y.); 2 School of Population and Public Health, University of British Columbia, 2206 East Mall, Vancouver, BC V6T 1Z3, Canada

**Keywords:** forest fire smoke, particulate matter, environmental exposure, asthma, public health surveillance, evaluation studies

## Abstract

The British Columbia Asthma Monitoring System (BCAMS) tracks forest fire smoke exposure and asthma-related health outcomes, identifying excursions beyond expected daily counts. Weekly reports during the wildfire season support public health and emergency management decision-making. We evaluated BCAMS by identifying excursions for asthma-related physician visits and dispensations of the reliever medication salbutamol sulfate and examining their corresponding smoke exposures. A disease outbreak detection algorithm identified excursions from 1 July to 31 August 2014. Measured, modeled, and forecasted concentrations of fine particulate matter (PM_2.5_) were used to assess exposure. We assigned PM_2.5_ levels to excursions by choosing the highest value within a seven day window centred on the excursion day. Smoky days were defined as those with PM_2.5_ levels ≥ 25 µg/m^3^. Most excursions (57%–71%) were assigned measured or modeled PM_2.5_ concentrations of 10 µg/m^3^ or higher. Of the smoky days, 55.8% and 69.8% were associated with at least one excursion for physician visits and salbutamol dispensations, respectively. BCAMS alerted most often when measures of smoke exposure were relatively high. Better performance might be realized by combining asthma-related outcome metrics in a bivariate model.

## 1. Introduction

British Columbia (BC) is the westernmost province in Canada. The region is heavily forested, and wildfires are a natural seasonal hazard that can threaten timber stocks, property, and air quality. With climate and land use changes, scientists are predicting more frequent fires, as well as longer and more severe fire seasons [1,2]. Since 1995, BC has experienced seven severe fire seasons, most of which broke records for the number of fires, hectares burned, and/or ambient temperatures [3]. Forest fires impact public health due to the threat of fire itself and through exposure to forest fire smoke. Epidemiologic studies have found that smoke exposure is associated with increased respiratory symptoms [4], dispensations of reliever medications for obstructive airway diseases [5,6], respiratory physician visits [7], hospital admissions [7,8], emergency department visits [9,10], and non-accidental mortality [11,12].

Following the severe fire season of 2010, BC medical health officers recommended the development of a province-wide passive surveillance system to track health effects associated with forest fire smoke. The BC Asthma Monitoring System (BCAMS) was established in 2012 by Environmental Health Services at the BC Centre for Disease Control (BCCDC) and provides near real-time surveillance of smoke and asthma-related health outcomes [13]. The objective of BCAMS is to support public health and emergency management decision-making by local health authorities.

The system was first piloted in 2012 and it has evolved with every fire season. In the first year we used daily dispensations of salbutamol sulfate as the indicator of asthmatic activity across the province. Salbutamol is the most common medication prescribed for acute relief of exacerbations of asthma or chronic obstructive pulmonary disease (COPD). The air quality impacts of forest fire smoke were assessed using fine particulate matter (PM_2.5_) measurements from the regulatory network. However, monitoring data are not available for all populated regions of the province, and this prompted us to develop a daily smoke exposure model that combined regulatory measurements with remote sensing data and meteorological forecasts [14] for use in future seasons.

In the summer of 2013 we started using the BCCDC Public Health Intelligence for Disease Outbreaks (PHIDO) alerting algorithm to identify days when salbutamol dispensation counts exceeded expected values based on historical trends, as discussed in the methods below. We also included information about forecasted PM_2.5_ concentrations from the BlueSky Western Canada Wildfire Smoke Forecasting System [15]. Previous work demonstrated that BlueSky forecasts were significantly associated with salbutamol dispensations and asthma-related physician visits in BC [16].

**Figure 1:**
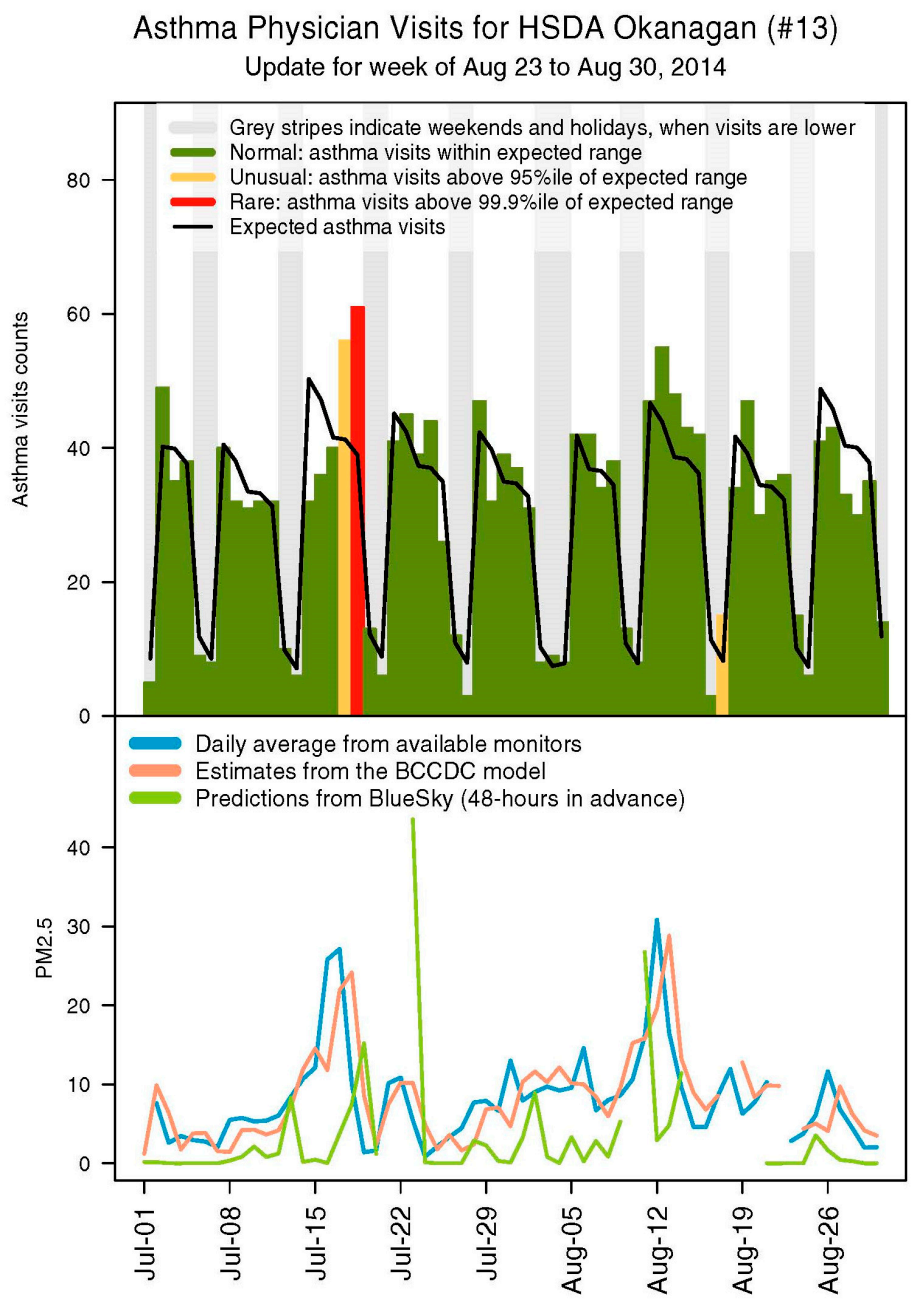
Sample Health Service Delivery Area (HSDA) summary page from a 2014 BC Asthma Monitoring System (BCAMS) report on outpatient physician visits. The upper panel shows expected daily counts of an asthma indicator, based on data from June 2012 onwards, and observed counts with any excursions highlighted. An alerting algorithm was used to identify excursions from the expected values, where counts over the 95th percentile of the expected distribution were considered unusual, and counts over the 99.9th percentile of the expected distribution were rare. The lower panel shows the different PM_2.5_ metrics. The plot for BlueSky shows the prediction made 48 h previously for the current day.

**Figure 2:**
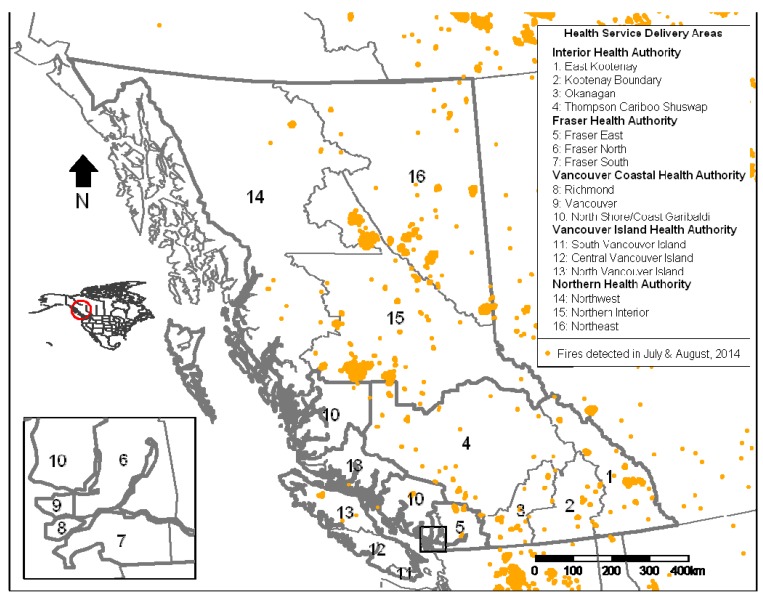
Health Service Delivery Areas in British Columbia and locations of fires during July and August, 2014 from the Hazard Mapping System at the United States National Oceanic and Atmospheric Administration.

Prior to 2014, pharmaceutical data were distributed weekly with a one-week lag. This changed to a two-week lag during the summer of 2014 for administrative reasons, so we adapted BCAMS to run with either dispensations of salbutamol sulfate or asthma-related physician visits from the provincial Medical Services Plan (MSP) records. Previous work demonstrated that smoke was significantly associated with increased asthma-related physician visits in BC [7,16,17] and these data are available daily. We continued to use measured, modeled, and forecasted PM_2.5_ concentrations through the summer and, at the end of the season, we received PM_2.5_ predictions from the FireWork forecasting model in development by Environment Canada [18]. As in other summers, we distributed a surveillance report (Figure 1) for the Health Service Delivery Areas (HSDAs) in each of the regional health authorities (Figure 2) weekly, or more frequently when there was significant fire activity.

The 2012 and 2013 forest fire seasons in BC were below average. In contrast, there were 369,002 hectares burned during the 2014 season, the largest area over the past decade [19,20]. In addition, the Northwest Territories experienced a very severe season in which 385 fires burned over three million hectares [21], and smoke from these fires impacted communities in BC and elsewhere in Canada. This level of activity during the 2014 season provides the first opportunity to meaningfully evaluate the performance of BCAMS and the utility of the different smoke exposure metrics it uses. Ultimately, public health practitioners want to know what smoke-related PM_2.5_ concentrations constitute a potentially harmful population exposure. To this end we designed the evaluation of BCAMS to answer two questions: (1) Do excursions reliably occur when PM_2.5_ concentrations are high? In addition, (2) At what PM_2.5_ concentrations can we reasonably expect to see a population response? To address these objectives we describe the numbers and types of excursions for salbutamol dispensations and asthma-related physician visits, and we examine how they were associated with the different PM_2.5_ metrics. We also calculate the sensitivity and specificity of various PM_2.5_ thresholds as a screening test for the presence of excursions.

## 2. Materials and Methods

### 2.1. Health Outcomes

Physician visit data were obtained from the BC Ministry of Health MSP billings database. These data are provided to the BCCDC daily, aggregated to the 16 provincial HSDA geographic units (Figure 2). Visits for asthma were identified using the International Classification of Diseases, 9th revision (ICD-9) code 493. Physicians submit claims to MSP on varying schedules that depend on several factors, including size of the practice. For instance, larger practices may submit billings daily, while smaller practices may submit billings weekly. Excursions were determined based on billings claimed within five days of the visit. Salbutamol dispensations were obtained from the BC PharmaNet database, which includes all prescriptions dispensed in the province by law [22]. The BC Ministry of Health provides these data to the BCCDC weekly, and there can be a two-week lag in the data they receive from the private contractor operating PharmaNet.

### 2.2. Exposure Variables

**Figure 3:**
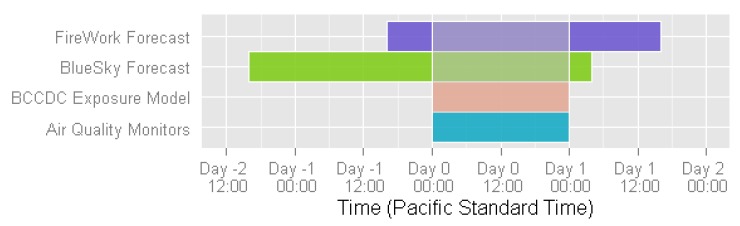
Exposure metrics timeline showing data used to calculate 24-h average exposure levels for Day 0.

Measured PM_2.5_ concentrations were obtained daily from the BC Ministry of Environment, and BCAMS uses 24-h averages calculated from midnight to midnight in Pacific Standard Time. If more than one monitoring station is located in an HSDA, measurements from all stations are averaged. The BCCDC forest fire smoke exposure model estimates daily PM_2.5_ using measured PM_2.5_ from the nearest monitoring station, aerosol optical depth and fire radiative power measured by satellite, hand-drawn smoke plumes traced from satellite images, and daily forecasts of the venting index from Environment Canada [14]. To assign a daily PM_2.5_ concentration to an HSDA, estimates at the geographic centres of census dissemination areas (DAs) from the 2006 census are selected. Population-weighted averages are then calculated using all estimates for the DAs within each HSDA. Forecasted PM_2.5_ concentrations were obtained from two smoke forecasting models: BlueSky [15], and FireWork [18]. The forecasts differ from measured and modeled PM_2.5_ in that they are specific to forest fire smoke. For BCAMS we use the forecasts predicted at 00:00 Coordinated Universal Time, and we use the DAs to calculate a population-weighted 24-h average prediction at the HSDA-level for each day based on the BlueSky forecast that was made two days in advance and the FireWork forecast that was made one day in advance (Figure 3).

### 2.3. Statistical Analysis

PHIDO is a disease outbreak detection algorithm developed at the BCCDC. It takes historical and current health outcome count data as input, and performs iterative regression using weighted generalized additive models to determine the expected distribution of the count data. The output identifies counts over the 95th, 99th, and 99.9th percentiles of the expected distribution. For BCAMS, counts over the 95th percentile of the expected distribution are considered unusual, and counts over the 99.9th percentile are rare. All statistical analyses and preparation of BCAMS reports were conducted in R 3.1.2 [23], and all code can be made available by the authors upon request.

### 2.4. Evaluation Objectives and Methods

To answer the two evaluation questions posed above, we: (1) summarized the excursions in asthma physician visits and salbutamol dispensations that occurred during the 2014 fire season; (2) determined levels of PM_2.5_ associated with each excursion; (3) determined the number of smoky days in each HSDA that had no associated excursions; and (4) calculated the sensitivity and specificity of different PM_2.5_ thresholds. We defined the 2014 forest fire season as starting 1 July 2014 and ending 31 August 2014. Although there were smaller fires burning in the province before and after this time period, it covered the largest and most intense fires.

We considered four categories of PM_2.5_ concentrations: 0–4.9, 5–9.9, 10–24.9, and 25+ µg/m^3^. The lowest two categories are the typical ranges of background PM_2.5_ in rural and urban areas of BC, respectively. Concentrations in the highest category exceed the World Health Organization 24-h mean guideline for PM_2.5_ of 25 µg/m^3^ [24]. To determine the concentrations of PM_2.5_ associated with each excursion, we considered all daily values that occurred within a 7-day window including the day of the excursion, the three days prior, and the three days following. We assigned the highest PM_2.5_ concentration during that time period to the excursion. Due to buildup of healthcare demand we removed excursions that occurred within the three days following a statutory holiday unless they were associated with a PM_2.5_ concentration in the highest category.

We defined smoky days as those when measured and/or modeled PM_2.5_ concentrations at the HSDA-level were ≥25 µg/m^3^ or when BlueSky and/or FireWork predicted a PM_2.5_ concentration ≥ 5 µg/m^3^. We used the same 7-day window to determine whether there was an excursion associated with a smoky day. For assigning PM_2.5_ concentrations to excursions and assigning excursions to smoky days, we included the three days before and after these occurrences to account for situations when excursions may precede the highest PM_2.5_ concentrations. For instance, PM_2.5_ levels may approach 25 µg/m^3^ for several days and an excursion can occur before this threshold is reached. An excursion could also occur before a spike in PM_2.5_ due to public health messaging encouraging susceptible populations to prepare for the event.

To calculate the sensitivity and specificity of different PM_2.5_ thresholds we used the following categories for each exposure metric: ≥5, ≥10, and ≥25 µg/m^3^. For each category we defined an “event day” as any day when PM_2.5_ was in the category of interest. Non-event days were days when PM_2.5_ was not in the category of interest. Non-event days that occurred within the 7-day window of an event day were removed from the analysis. If an excursion was found within the 7-day window of an event day, this was considered a true positive (TP). If no excursion was found within the window of a non-event day, this was considered a true negative (TN). A false positive (FP) was counted if a non-event day had an associated excursion, and a false negative (FN) was counted if an event day did not have an associated excursion. The sensitivity (TP/TP + FN) and specificity (TN/TN + FP) were calculated for excursions of asthma-related physician visits and salbutamol dispensations.

## 3. Results and Discussion

### 3.1. Summary of Excursions

There were 35 excursions for asthma-related physician visits during the 2014 fire season, after excluding six that occurred within three days after statutory holidays (Table 1). The Interior and Northern health authorities had the most excursions with 11 and eight, respectively. There was only one rare excursion, and this occurred in the Interior. There were 48 excursions for salbutamol dispensations, after excluding 12 that occurred close to statutory holidays (Table 1). Again, the Interior and Northern health authorities had the most excursions with 15 and 13, respectively, and all nine rare excursions occurred in these regions.

**Table 1:** Summary of excursions for asthma physician visits and salbutamol dispensations.

Health Authority	Asthma Visit Excursions	Unusual Excursions (%)	Rare Excursions (%)	Salbutamol Dispensation Excursions	Unusual Excursions (%)	Rare Excursions (%)
**Fraser**	7	7 (100)	0 (0.0)	5	5 (100)	0 (0.0)
**Interior**	11	10 (90.9)	1 (9.1)	15	10 (66.7)	5 (33.3)
**Northern**	8	8 (100)	0 (0.0)	13	9 (69.2)	4 (30.8)
**Vancouver Coastal**	3	3 (100)	0 (0.0)	8	8 (100)	0 (0.0)
**Vancouver Island**	6	6 (100)	0 (0.0)	7	7 (100)	0 (0.0)
**Total**	**35**	**34 (97.1)**	**1 (2.9)**	**48**	**39 (81.2)**	**9 (18.8)**

### 3.2. PM_2.5_ Levels Associated with Excursions

#### 3.2.1. Measured PM_2.5_ from Monitoring Stations

**Figure 4:**
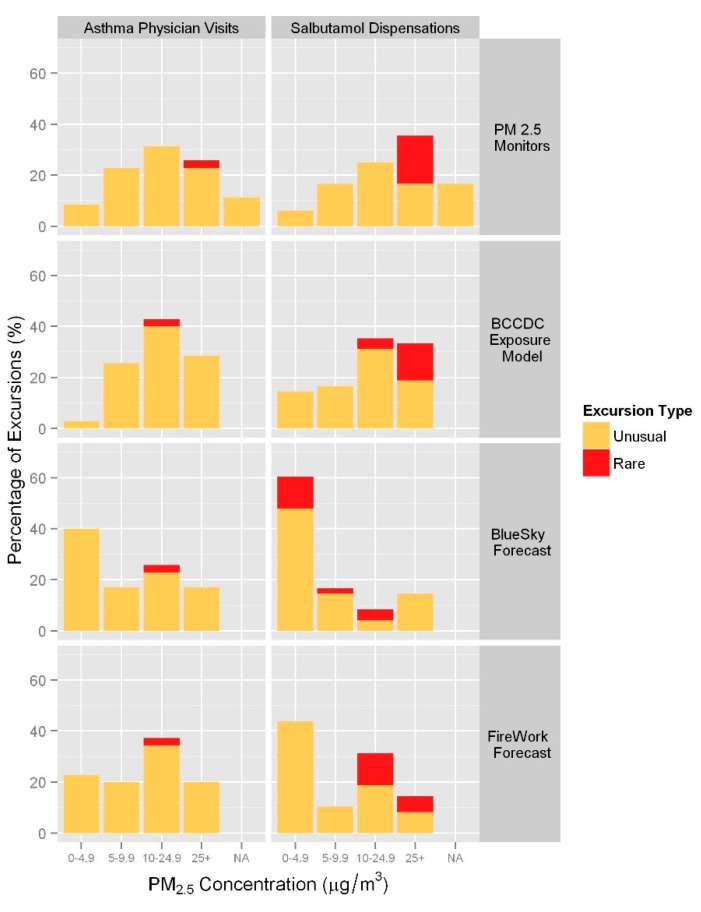
Percentage of excursions by highest PM_2.5_ level (µg/m^3^) within the seven-day window for each exposure metric.

The highest measured PM_2.5_ concentrations assigned to excursions ranged from 3.4 to 75.8 µg/m^3^. A measured PM_2.5_ level was not available (NA) for 11.4% of asthma physician visit excursions and 16.7% of salbutamol dispensation excursions due to missing data. For the asthma-related physician visits, 55.9% of the unusual excursions were associated with levels ≥10 µg/m^3^, and 100% of the rare excursions were associated with levels ≥25 µg/m^3^. Similarly for salbutamol dispensations, 51.3% of the unusual excursions were assigned levels ≥10 µg/m^3^, and 100% of the rare excursions were assigned levels ≥ 25 µg/m^3^ (Figure 4).

#### 3.2.2. Modeled PM_2.5_ from the BCCDC Forest Fire Smoke Exposure Model

The highest modeled PM_2.5_ concentrations associated with excursions ranged from 3.0 to64.4 µg/m^3^. Compared with measured PM_2.5_, more of the unusual asthma-related physician visit excursions (70.6%) were assigned modeled PM_2.5_ levels ≥10 µg/m^3^, but the one rare excursion was assigned a level in the 10–25 µg/m^3^ range (Figure 4). Modeled PM_2.5_ levels were ≥10 µg/m^3^ for 61.5% of unusual salbutamol dispensation excursions, and ≥25 µg/m^3^ for 77.8% of rare excursions.

#### 3.2.3. Forecasted PM_2.5_ from BlueSky and FireWork

The BlueSky and FireWork PM_2.5_ predictions associated with excursions ranged from 0 to 51.2 µg/m^3^ and 0 to 76.1 µg/m^3^, respectively. Compared with measured and modeled PM_2.5_, the majority of excursions fell within the 0–9.9 range of the smoke-specific PM_2.5_ forecast values (Figure 4). Rare asthma-related physician visit excursions were associated with BlueSky and FireWork predictions in the 10–24.9 µg/m^3^ range. Rare salbutamol dispensation excursions were associated with BlueSky predictions in the lower three categories (0–24.9 µg/m^3^), and FireWork predictions in the highest two categories (≥10 µg/m^3^).

### 3.3. Smoky Days and Occurrence of Excursions

There were 43 HSDA days during the forest fire season when daily measured and/or modeled PM_2.5_ levels were ≥25 µg/m^3^ (Table 2), from a possible total of 992 days (62 days in the study period times 16 HSDAs). These observed smoky days were distributed among 6 six HSDAs in the Interior and Northern health authorities. In 24 cases (55.8%), at least one asthma physician visit excursion occurred within the seven-day window. For salbutamol dispensations, 30 observed smoky days (69.8%) were associated with at least one excursion.

BlueSky and/or FireWork smoke-specific PM_2.5_ concentrations were ≥5 µg/m^3^ on 280 days during the fire season, distributed among all 16 HSDAs. For 66.1% and 72.5% of forecasted smoky days there were no associated asthma physician visit or salbutamol dispensation excursions, respectively.

**Table 2:** Summary of days with measured and/or modeled PM_2.5_ levels of 25 µg/m^3^ or higher and the occurrence of excursions in asthma physician visits and salbutamol dispensations. Of the 16 HSDAs, 10 are not included in the table because they had no days meeting these criteria.

Health Service Delivery Area	Total Days with PM_2.5_ ≥ 25 µg/m^3^ (% of Study Period)	Not Matched to Any Asthma Visit Excursion	Matched to an Asthma Visit Excursion	Not Matched to Any Salbutamol Dispensation Excursion	Matched to a Salbutamol Dispensation Excursion
East Kootenay	3 (4.8)	0	3	0	3
Kootenay Boundary	3 (4.8)	3	0	1	2
Okanagan	4 (6.4)	2	2	2	2
Thompson Cariboo Shuswap	7 (11.3)	3	4	3	4
Northern Interior	14 (22.6)	4	10	0	14
Northeast	12 (19.3)	7	5	7	5
Total	43 (4.3)	19	24	13	30

### 3.4. Screening Test Parameters

The sensitivity of a PM_2.5_ threshold indicates the proportion of days over that threshold on which an excursion occurred within the seven-day evaluation window. Conversely, the specificity indicates the proportion of days under that threshold on which an excursion did not occur. A threshold that approaches 100% for both values suggests the PM_2.5_ concentration at which we can reasonably expect to see a population response. Our calculated sensitivities ranged from 25.1% for asthma-related physician visit excursions associated with measured PM_2.5_ levels ≥ 5 µg/m^3^ to 85.7% for salbutamol dispensation excursions associated with measured PM_2.5_ levels ≥ 25 µg/m^3^ (Table 3). In general, the sensitivity increased as the lower bound for measured and modeled PM_2.5_ increased, but the pattern was less consistent for forecasted PM_2.5_. The specificities were consistently around 90% for asthma-related physician visits and 80% for salbutamol dispensations for all thresholds of all exposure metrics. The ≥25 µg/m^3^ threshold was clearly the best for the measured and modeled exposure metrics, but the sensitivities for forecasted values were less than 40% in 11 of 12 cases.

**Table 3:** Sensitivity and specificity of different PM_2.5_ thresholds for indicating the presence of an excursion. The best threshold (as measured by the sum of sensitivities and specificities across each row) for each exposure metric and each health outcomes is highlighted in bold.

PM_2.5_ Exposure Metric	PM_2.5_ Exposure Category	Sensitivity for Asthma-Related Physician Visits	Specificity for Asthma-Related Physician Visits	Sensitivity for Salbutamol Dispensations	Specificity for Salbutamol Dispensations
Monitor	≥5 µg/m^3^	25.1	84.2	27.0	80.3
	≥10 µg/m^3^	35.7	91.5	41.6	86.5
	**≥25 µg/m^3^**	**71.4**	**91.0**	**85.7**	**85.3**
Model	≥5 µg/m^3^	27.4	92.2	26.8	68.6
	≥10 µg/m^3^	34.7	91.3	32.4	83.0
	**≥25 µg/m^3^**	**58.1**	**89.8**	**67.7**	**84.0**
BlueSky	≥5 µg/m^3^	28.1	90.7	22.9	81.7
	≥10 µg/m^3^	32.7	91.5	23.1	81.9
	**≥25 µg/m^3^**	**30.8**	**90.4**	**46.2**	**85.0**
FireWork	≥5 µg/m^3^	34.4	92.1	27.9	82.7
	**≥10 µg/m^3^**	**34.8**	**90.8**	**31.2**	**83.9**
	≥25 µg/m^3^	37.8	90.0	27.0	84.0

### 3.5. Discussion

In this evaluation of BCAMS, we summarized excursions for asthma-related physician visits and salbutamol dispensations, examined corresponding smoke exposures, considered smoky days not associated with any excursions, and calculated the sensitivity and specificity of different PM_2.5_ thresholds. We found that most excursions were associated with relatively high measured or modeled PM_2.5_ concentrations (≥10 µg/m^3^) compared with the typically low median fire season exposures ranging from 4 µg/m^3^ (Vancouver Island Health Authority) to 7.1 µg/m^3^ (Fraser Health Authority). In addition, we found that the highest numbers of excursions and all observed smoky days occurred in the Interior and Northern Health Authorities. These regions were most affected by fires, encompassing 87.7% of the total fires and 99.6% of the total hectares burned [20]. Our results suggest that asthma-related physician visits and salbutamol dispensations are suitable for monitoring the population health impacts of forest fire smoke, and that the exposure metrics are capturing smoke events when they occur.

The BlueSky and FireWork forecasts are specific to PM_2.5_ from forest fires, rather than reflecting PM_2.5_ from all sources. In contrast to measured and modeled PM_2.5_, we found that the majority of excursions for both outcome measures were associated with forecasted PM_2.5_ concentrations < 10 µg/m^3^. This finding has two possible interpretations. One is that the presence of any forest fire smoke affects asthma-related health outcomes at the population level. A second possibility is that this finding reflects the significant challenges involved in forecasting smoke.

To further evaluate the utility of the different exposure metrics for indicating PM_2.5_ concentrations at which we can reasonably expect to see a population response, we calculated the sensitivity and specificity of various PM_2.5_ thresholds for each. The specificity was generally high across all thresholds for all metrics, reflecting the fact that most days had low PM_2.5_ concentrations and no health outcome excursions. The sensitivity was >70% for both health outcomes when measured PM_2.5_ concentrations were ≥25 µg/m^3^, but there was no clear threshold for PM_2.5_ predictions from either smoke forecasting system. This analysis was not a typical sensitivity and specificity analysis involving a simple clinical outcome with a recognized medical gold standard. Instead we have used sensitivity and specificity as tool to explore how different PM_2.5_ thresholds indicate the presence of an excursion.

Although both of the health outcomes used in BCAMS reflect exacerbations of asthma in the population, we expect them to respond differently to smoke exposure. In general, more people will refill their medications in response to an exacerbation than will visit their physicians and, as a result, we expect to see a stronger signal with salbutamol dispensations during smoky periods. It is also possible that the signal for dispensations is more timely, because a prescription can be filled promptly whereas a patient may have to wait to see a physician. In fact, we did see a greater number of salbutamol dispensation excursions (48) over the fire season compared with asthma-related physician visit excursions (35), suggesting that dispensations are more common. There were also more rare salbutamol dispensation excursions (18.8%) compared with rare physician visit excursions (2.9%). Similarly, observed smoky days were more frequently associated with salbutamol dispensation excursions (69.8%) than with physician visit excursions (55.8%). These are both evidence of a slightly stronger signal for salbutamol dispensations, and perhaps a more timely signal in the case of high smoke exposure.

Excursions for asthma-related physician visits and salbutamol dispensations can occur for reasons other than forest fire smoke, which appear as random noise in the data. This is evidenced by the fact that we saw excursions associated with a range of PM_2.5_ levels for all exposure metrics. Some of this noise was reduced by removing excursions immediately following statutory holidays. Similarly, we chose to consider conditions before and after excursions and smoky days to account for reverse-causation where asthmatics may refill their medication or visit their physician in response to public health messaging before or during a smoke event. The BCAMS reports explain that excursions are not always related to air quality issues, and the summary pages include visual information on both health outcomes and exposure so that users can disregard excursions not associated with higher PM_2.5_ exposure. Interestingly, observed smoky days not associated with any excursions occurred more often during August for both asthma physician visits (78.9% were in August) and salbutamol dispensations (100% were in August). This suggests that susceptible populations were more prepared for periods of smoke exposure later in the fire season.

There are two important limitations in BCAMS. The first involves timeliness of the respiratory data sources. As noted above, the MSP billings database is populated over time as physicians submit their billings, and we determined excursions based on billings claimed within five days of the visit. This means that BCAMS reports based on asthma-related physician visits are not available until five days following any given date. Although the PharmaNet database is populated daily, BCAMS reports based on salbutamol dispensations are not available for up to two weeks following any given date due to the schedule on which these data are released by the private contractor operating PharmaNet. This significant lag precludes the use of these data in a near real-time surveillance system. At this time, BCAMS produces weekly reports covering the recent past to help BC public health professionals understand what has already occurred in fire-affected regions. If truly real-time health data become available, BCAMS could better inform the public health response by providing early warning of population health effects during fire events.

The second limitation involves potential misclassification of health outcomes and exposure estimates. Using diagnosis codes introduces uncertainty because physicians can have differing coding practices. Exposure to forest fire smoke is difficult to estimate as levels of smoke can vary rapidly over time and space. Aggregating exposure estimates over large areas introduces exposure misclassification where those living in a given area are assigned a higher or lower exposure than what they truly experience. One advantage with the PharmaNet data is that salbutamol dispensations are available aggregated to the smaller Local Health Area (LHA) geographic units, allowing comparisons with exposure estimates aggregated over smaller areas. There are insufficient asthma-related physician visits on a daily basis in some LHAs to permit aggregation at this level considering both data privacy issues and statistical robustness.

There are few examples in the literature of passive surveillance systems like BCAMS focused on forest fire smoke exposure and health outcomes. During the California wildfire event in 2003, San Diego County Public Health used existing surveillance resources to monitor over-the-counter medication sales, emergency department visits and paramedic transport data in addition to air quality data [25]. Similarly, during another severe wildfire event in 2007, passive surveillance of emergency department visit data in Los Angeles County supported the decision to declare a smoke advisory [26]. Surveillance of emergency department data was also conducted during this event in San Diego County using the Centers for Disease Control and Prevention’s BioSense System [27], and data from both the 2003 and 2007 events have been used to model the future impact of wildfires on respiratory health given changing climate conditions [28]. This work adds to these previous examples as BCAMS uses different health outcomes and a wider range of exposure metrics.

## 4. Conclusions

In conclusion, we evaluated the performance of BCAMS during the 2014 forest fire season and found that the system alerted most often when measures of smoke exposure were relatively high. Salbutamol dispensations and asthma-related physician visits contribute slightly different, but complimentary, surveillance information. BCAMS will continue to evolve over the coming years. Methods are currently being explored to simultaneously model both health outcomes, rather than treating them separately. This approach may provide more information so that the BCAMS product can offer better support to those making challenging public health decisions during forest fire events.
